# Is frailty associated with increased concerns about falling and activity restriction in community-dwelling older adults? A systematic review

**DOI:** 10.1016/j.tjfa.2024.100002

**Published:** 2025-01-01

**Authors:** Bianca Nicklen, Kim Delbaere, Toby J. Ellmers

**Affiliations:** a Department of Brain Sciences, Imperial College London, UK; b Falls, Balance and Injury Research Centre, Neuroscience Research Australia, Randwick, NSW, Australia; c Australia and School of Population Health, University of New South Wales, Kensington, NSW, Australia

**Keywords:** Falls, Activity avoidance, Fear of falling, Anxiety, Older adults

## Abstract

**Purpose:**

Concerns about falling (CaF) are common in older adults. They are associated with increased risk of falls, activity restriction, social isolation, and physical deconditioning. This systematic review assessed if frailty is a risk factor for CaF.

**Methods:**

Searches of cross-sectional and prospective studies exploring associations between frailty and CaF were conducted across five databases (Medline, CINAHL, Embase, Psychinfo and Scopus). The Risk of Bias in Non-randomised Studies of Exposure (ROBINS-E) was used to determine risk of bias.

**Results:**

The search identified 2492 articles, 12 were included for data extraction: 8 cross-sectional and 4 prospective studies. Participants’ mean ages across the different studies ranged from 67.5 – 81.7 years. All adjusted analyses reported a significant association between increasing frailty and CaF, except for one cross-sectional paper. Significant adjusted Odd Ratios (ORs) ranged from 1.79 (CI = 1.18-2.71) to 144.78 (CI = 13.86 – 1512.60) for cross-sectional studies, and from 1.33 (CI = 1.04–1.69) to 12.4 (CI = 7.6-20.1) for prospective studies. Three studies (one cross-sectional and two prospective) explored the association between frailty and concern-related activity restriction: A significant association was reported in two prospective studies (adjusted OR = 1.58 (CI=1.09-2.30) and adjusted RRR = 3.91 (2.61-5.85)), but not the cross-sectional study (adjusted OR = 1.31 (CI=0.62-2.78)).

**Conclusion:**

This review identifies strong associations between increasing frailty and both CaF and associated activity restriction. This expands previous work describing the opposite association (that CaF can lead to frailty), suggesting a bi-directional relationship. Clinicians working with pre-frail and frail older adults should consider screening for CaF.

**Prospero:**

CRD42023371899.

## 1 Introduction

Population ageing is increasing globally, with projections indicating that 25 % of the world's population will be aged 65 and above by 2050 [1]. Ageing is associated with an increase in comorbidities and geriatric syndromes, such as frailty [2]. Frailty denotes a dynamic multisystem decline of different physiological reserves, culminating in a reduced capacity to respond to external stressors and a subsequent increased risk of adverse health outcomes [[2], [3], [4]]. Prevalence rates range from 3.9 % to 51.4 %, contingent on the screening tool used, as well as the population being assessed (e.g. community dwellers, nursing home residents, inpatients) [4]. Frailty is associated with a reduction in activities of daily living, as well as increased incidence of falls, fractures, hospitalisation and death [5,6]. Risk factors include female gender, poor nutrition, low socioeconomic background, functional disability, hospitalisation within the last year, previous falls, advanced age, vision problems, cognitive impairment, comorbidities, and polypharmacy [[7], [8], [9], [10]]. Psychological factors such as anxiety, depression and concerns (or, “fears”) about falling (CaF) have also been identified as potential risk factors [11,12].

CaF are common in older adults [13], and refer to “a lasting feeling of dread and apprehension about situations that are believed to threaten or challenge balance” [14]. Notably, the Word Falls Guidelines highlights that whilst the term ‘fear of falling’ is widely used in peer-reviewed literature, utilising ‘concerns’ over ‘fear’ holds many advantages [15]. This is because ‘fear’ implies an emotional experience akin to a phobia which may or may not be accurate, and the emotionality of the construct may discourage older individuals from disclosing any such experiences. In contrast, ‘concerns’ implies a less intense and emotional construct [16]. This article will therefore utilise the term ‘concerns about falling’ as an umbrella term that encapsulates ‘fear of falling’ and all other similar terminology relating to the psychological outcome of falls (e.g., worries or anxiety about falling). The World Falls Guidelines recommended using the Falls Efficacy Scale – International (FES-I) as the ‘gold standard’ method for assessing CaF [15].

Although some degree of concerns may be adaptive, particularly if these reflect a realistic appraisal of an individual's risk of falling [14], CaF are often maladaptive. They are associated with various negative outcomes, including depression, functional decline and reduced quality of life [13,[16], [17], [18], [19]]. CaF can also often lead to activity restriction, especially outside the home, due to an increased perceived risk of falling [20,21]. This association might explain how CaF can increase the risk of frailty, with CaF-related activity restriction leading to greater physical deconditioning [11]. A recent qualitative paper described how CaF often develop in response to an individual recognising both their ‘ageing body’ and risk for experiencing a ‘serious’ fall [22]. This therefore also suggests a potential bi-directional relationship between CaF and frailty, indicating that frailty might also be a risk factor for – and not just a consequence of – CaF. Emerging evidence supports this notion: Makino et al. reported that baseline frailty was independently associated with greater odds of having CaF at follow-up [23], and other studies have also reported that interventions aimed to target frailty can also have secondary effects on CaF [24,25].

While a recent systematic review determined that CaF was a risk factor for frailty [11], the converse relationship – i.e., whether frailty is a risk factor for CaF – has not yet been reviewed. Developing a better understanding of the relationship between frailty and CaF holds clinical relevance, enabling early identification of those most at risk of CaF, and thereby facilitating timely and effective treatment to prevent the associated negative outcomes. Therefore, the aim of this systematic review is to evaluate whether increasing frailty is associated with greater CaF (and associated activity restriction) in older adults.

## 2 Method

### 2.1 Design protocol and registration

The current study conformed with the Preferred Reporting Items for Systematic Reviews and Meta-Analysis (PRISMA [26]) while following the Population, Intervention, Comparison and Outcomes (PICO) framework [27]. The protocol was registered in the International Prospective Register of Systematic Reviews: PROSPERO, registration number: CRD42023371899.

### 2.2 Literature search strategy

The publication date of the studies was not an eligibility criterion, and all searches were conducted in December 2022 (and updated in March 2024) on five electronic databases: Medline, CINAHL, Embase, Psychinfo and Scopus. Additional relevant literature obtained from other sources was also added to the PRISMA flow chart.

Search terms and keywords were conducted according to the PICO framework and were combined with “AND”. The main keywords included: population “older adults”, “aged”, “elderly”; intervention/exposure “frailty”; outcome “concerns about falling”, “fear of falling”, “activity restriction”. Full keywords and search terms can be found in Appendix 1. All searches were exported into Covidence [28] for processing according to the PRISMA flowchart [26].

### 2.3 Eligibility criteria

We focused on studies in which the main objective was to assess whether frailty is associated with CaF and/or CaF-related activity restriction. Although longitudinal prospective studies provide a stronger evidence base for a causal relationship between frailty and CaF, we also chose to include cross-sectional studies to capture a broader range of evidence and ensure that findings are consistent across different study designs. Studies that met the following inclusion criteria were included: 1) Published in English; 2) participants could be both community-dwelling and also residing in institutions (e.g. residential care homes, assisted care facilities, sheltered housing or retirement communities); 3) average age of participants was over 65 years old^1^, 4) the inclusion of a validated multi-component measurement of frailty as a predictor outcome (e.g. the article did not just include weakness or slowness alone as predictors); 5) CaF and/or CaF-related activity restriction as an outcome variable. To focus on activity restriction due to CaF, papers had to directly assess CaF-related activity restriction through patient-reported measurement (e.g. asking “do you ever avoid activities due to being concerned that you might fall?”).

Articles were excluded if they: 1) were not cross-sectional or prospective, including intervention studies, other systematic reviews or meta-analysis, case studies, and qualitative studies; 2) recruited participants specifically on the basis of disease status/health conditions other than frailty (e.g., cognitive impairment or Parkinson's Disease); 3) studied individuals who were currently residing in an acute care setting (e.g. in-patient hospital); 4) if the authors did not conduct a regression analysis with frailty predicting CaF (either in the original paper or upon request).

### 2.4 Selection of studies for review and data extraction

Two reviewers (B.N. and T.E.) independently reviewed all the articles in two stages. First, all searches from electronics databases were imported into Covidence where duplicates were highlighted, before being manually checked and then removed. The two reviewers then separately screened all titles and abstracts, followed by full text screening. At each stage, articles that did not meet the inclusion criteria were excluded, and any disagreements between reviewers were discussed and resolved.

Data from the final articles were extracted by the lead author (B.N.) and then confirmed for accuracy by a second author (T.E.). Extracted information included: location and setting of the study, the assessment tools used to assess frailty and CaF, and the main findings from the study (including, adjusted and unadjusted odds ratios, 95 % confidence intervals and p-values where provided). Due to the marked heterogeneity observed across the included studies (with respect to how frailty and CaF were assessed and determined), it was not possible to pool results into a meta-analysis.

### 2.5 Risk of Bias assessment

To assess the risk of bias for all the included studies, the two reviewers (B.N. and T.E.) independently used the Risk of Bias in Non-randomised Studies of Exposure (ROBINS-E [29]). The ROBINS-E tool assessed each paper for seven areas of bias associated to: confounding, the selection of participants into the study, the classification of exposure, the deviation from intended exposure, missing data, the measurement of outcome and in the selection of reported results. Studies were rated as having a low risk of bias on the confounding domain if they controlled for two or more of the following variables: age, gender or previous falls. All subcategories were then assigned with either: “low risk of bias”, “moderate risk of bias”, “serious risk of bias”, “critical risk of bias” or “no information”. An overall rating of risk of bias was then given to each paper.

## 3 Results

### 3.1 Study selection

**Fig. 1:**
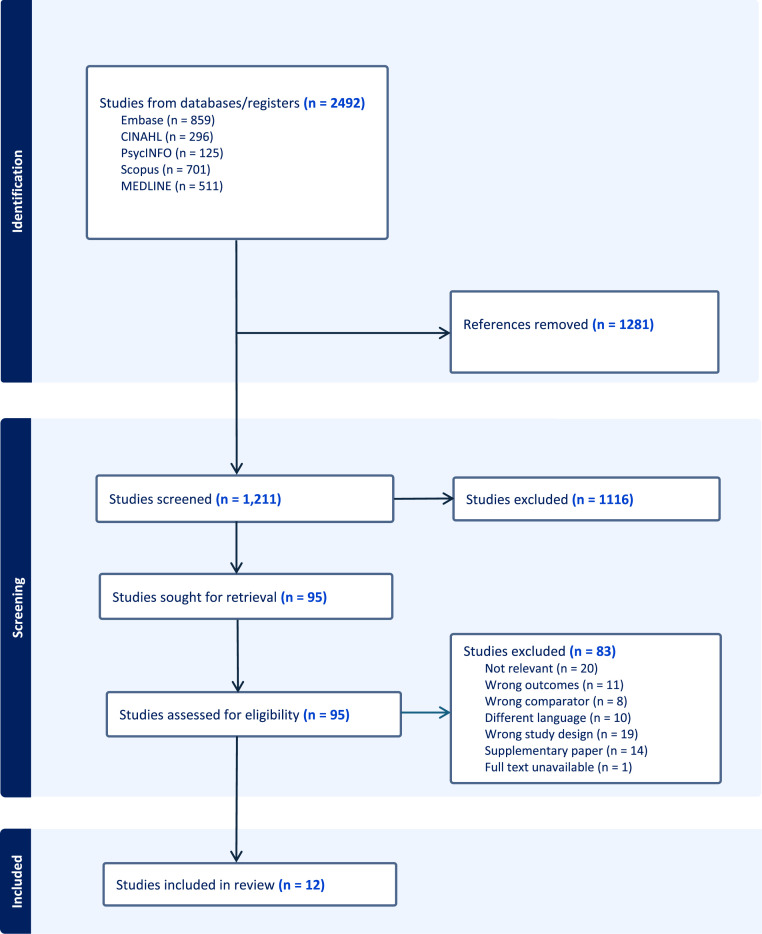
Full screening PRISMA flowchart.

The initial searches from the five databases yielded 2492 articles for review. Following the removal of 1281 duplicates, 1211 articles remained for title and abstract screening. Ninety-five articles were assessed for full-text screening, whereby a further 83 were excluded. This resulted in a final list of 12 papers [23,[30], [31], [32],[33], [34], [35], [36], [37], [38], [39], [40]]. Fig. 1 displays the PRISMA flow chart detailing the full screening process.

### 3.2 Study characteristics

There were eight cross-sectional [[30], [31], [32],[33], [34], [35], [36],^39^] and four prospective studies [23,37,38,40]. In total, there were 17,978 participants included across the 12 studies, with sample sizes of each individual study ranging between 183 [34] to 5829 [38]. The sample total was 59.3 % female and 40.7 % male, with every included study containing a greater proportion of females to males. The average ages for 11 studies were between 67.5 and 81.7, with one paper [29] not reporting their overall mean ages (despite attempts to contact the authors for this information) but instead stating that each participant was aged between 65-74 years.

The participants were all community-dwelling older adults, and data were collected in various countries, including: Singapore (n = 2 [31,35]); Spain (n = 2 [33,34]); Japan (n = 1); Brazil (n = 1 [36]); India (n = 1 [30]); Turkey (n = 1 [32]); England (n = 1 [40]); United States (n = 1 [38]). Two studies reported data collected in multiple countries: Curcio et al. [37] reported data collected in Albania, Brazil, Canada, and Colombia, and Valsecchi et al. [39] reported data collected in Spain and the Netherlands.

### 3.3 Frailty assessment tool

Frailty was measured using five different assessment tools: Eight studies used the Fried frailty index (FFI) [[3], [23], [30], [33], [34], [36], [37], [38], [40]]; two used the 5-item FRAIL scale [31,32]; De Roza et al. [35] used the Clinical Frailty Scale (CFS [4]), Valsecchi et al. [39] used the Tilburg Frailty Indicator (TFI) [41], and Kendhapedi and Devasenapathy [30] used both the Frailty Index (FI [42]) and the TFI.

There was not a unanimous categorisation of frailty between papers. Non-frail was compared to the combined category of pre-frail and frail in five studies [23,31,32,33,38]. One study combined non-frail and pre-frail, and compared this categorisation to frail [34]. Four studies classified frailty level into three or more categories (e.g., robust vs. pre-frail vs. frail) [30,[35], [36], [37]], and two studies looked at frailty as a continuous variable [39,40]. Further, the proportion of participants who were robust, pre-frail and frail varied greatly between studies. For example, incidence of frailty as determined by the most frequently used FFI varied between 8.0 % [36] to 53.6 % [34]. Table 1 shows further breakdown of the specific frailty assessments used in each study, including the number of participants in each frailty category.

**Table 1:** Details about the measurement tools and prevalence of frailty and CaF in the individual studies.

	Frailty measurement tool	Robust (%)	Prefrail (%)	Frail (%)	CaF measurement tool	Overall prevalence of CaF (%)	
**Cross-sectional**							
De Roza et al., 2022	CFS	29.4	51.4	19.2	Short FES-I	60.8	
De Souza Moreira et al., 2017	FFI	39.7	52.3	8.0	FES-I	53.6	
Esbri-Victor et al., 2017	FFI	46.4		53.6	Three single-item questions to determine CaF; FES-I	CaF = 76.5	High FES-I = 79.2
Kendhapedi & Devasenapathy 2019	FFI	23	49.2	27.6	Short FES-I	51.7	
	FI	41.25		58.8			
	TFI	37.4		62.6			
Merchant et al., 2020^a^	5-item FRAIL	48.7	51.3		Single-item question	CaF = 69.2	CaF+AR = 38.4
Ozturk et al., 2021	5-item FRAIL	36.3	39.6	18	Single-item question	44.6	
Perez-Ros et al., 2020	FFI	16.1	83.9		Modified FES-I	Overall prevalence not reported	
Valsecchi et al., 2023	TFI	Continuous variable (prevalence data not reported)			FES-I	Overall prevalence not reported	
**Prospective**							
Curcio et al., 2020	FFI	47.8	45.7	6.5	FES-I	24.8	
Ellmers et al., 2023^b^	FFI	24.1	57.5	18.4	Single-item question	CaF+AR = 10.1	
Makino et al., 2021	FFI	49.0	51.0		Single-item question	41.5	
Mo et al., 2023^b^	FFI	36.1	48.7	15.2	Single-item question	CaF without AR = 16.7	CaF+AR = 11.9

CaF = Concerns about falling. CaF+AR = activity restriction due to concerns about falling. CFS = Clinical Frailty Scale. FES-I = Falls Efficacy Scale-International. FFI = Fried Frailty Index. FI = Frailty Index. TFI = Tilburg Frailty Indicator.

a This study looked at both CaF and activity restriction due to CaF as an outcome variable.

b This study looked at activity restriction due to CaF as the outcome variable, rather than CaF alone.

### 3.4 Concerns about falling assessment tool

The overall prevalence of CaF was reported by all papers except for Perez-Ros et al. [33] and Valsecchi et al. [39]. Overall prevalence rates can be found in Table 1. CaF were dichotomised into present or absent (or high or low) in all studies except for two, one which split CaF into three categories (low, moderate, and high) [30] and the other that used CaF as a continuous variable [39].The full 16-item FES-I was used in four studies [34,36,37,39], the short 7-item FES-I was used in 2 studies [30,35], and one paper reported using a modified FES-I [33]. Five studies used a single-item question [23,31,32,38,40], whilst Esbri-Victor et al. asked three separate single-item questions to determine CaF. They were also the only authors to compare two separate CaF assessment tools: the FES-I, as well as three closed questions (which encompassed both CaF and associated activity restriction). Three papers additionally measured activity restriction due to CaF using single-item questions [31,38,40].

### 3.5 Outcomes

All extracted outcome data for cross-sectional and prospective studies can be found in Table 2, Table 3, respectively. The unadjusted OR for cross-sectional studies varied between 4.78 (CI = 2.22-10.27) to 144.78 (CI = 13.87-1512.6). For prospective studies, unadjusted ORs ranged from 1.43 (CI = 1.15-1.79) to 12.4 (CI = 7.6**-**20.1).

**Table 2:** Outcomes from cross-sectional studies.

Study	Location & Setting	Sample size; Gender (male (M) and female (F) (%))	Age	Frailty assessment tool and cut-offs used in analysis	Concern about Falling (CaF) assessment tool and cut-offs used in analysis	Main findings: With frailty status/level as the predictor, CaF presence/severity as the outcome variable, odds ratios (OR) (adjusted and unadjusted), 95 % confidence interval (CI), p value
De Roza et al., 2022	Singapore; Community-dwelling	360 M: 145 (40.3) F: 215 (59.7 %)	Mean = 78.3	CFS Well vs. pre- vs. mildly- vs. moderately-frail **Cut-offs used:** - Well: scores 1-3 - Pre-frail: score 4 - Mildly Frail: score 5 - Moderately frail: score 6 (Scores ≥ 7 excluded)	Short FES-I; **Outcome:** High (≥14/28) vs. low/moderate concerns (<14/28)	Frailty (compared to well) have greater odds of high CaF: Pre frail vs. well: **unadjusted OR** = 6.87 (CI = 2.66-17.74), p < .001); Mildly frail vs. well: **unadjusted OR** = 18.58 (CI = 4.88 – 70.74), p < .001); Moderately frail vs. well: **unadjusted OR** = 144.78 (CI = 13.86 – 1512.60), p < .001).
De Souza Moreira et al., 2017	Brazil; Community-dwelling	3594 (non-diabetic subset of the sample) M: 1217 (33.9 %) F: 2377 (66.1 %)	Mean = 73.5 ± 6.5	FFI Robust vs. pre-frail vs. frail **Cut-offs used:** - Robust: none of the criteria - Pre-frail: one or two criteria - Frail: three or more criteria	FES-I; **Outcome:** Presence (≥23/64) vs. absence (<23/64) of CaF	Pre-frail and frail, respectively, compared to non-frail have higher odds of high CaF: Pre-frail vs. robust: adjusted OR* = 1.16 (CI = 0.96-1.39), p = .120 Frail vs. robust: adjusted OR* = 1.79 (CI = 1.18-2.71), p = .006 *adjusted for gender, arthritis/rheumatism, depression, visual impairment, negative health self-perception, falls in previous 12 months, being overweight, underweight or obese, depressive symptoms, Katz Index (ADL), Lawton Scale (IADL), handgrip strength, gait speed.
Esbri-Victor et al. 2017	Spain; Community-dwelling	183 M: 36 (19.7 %) F: 147 (80.3 %)	Mean = 78.4 ± 5.6	FFI Robust + prefrail vs. frail **Cut-offs used:** - Robust: none of the criteria - Pre-frail: one or two criteria - Frail: three or more criteria	Three yes/no questions: 1) Are you afraid of falling?; 2) Do you limit any household activities because you are frightened you may fall?; 3) Do you limit any outside activities because you are frightened you might fall? **Outcome:** Presence of CaF (positive answer to any of the three questions) vs. absence of CaF.	Frailty vs. pre-frail or robust was associated with increased odds of CaF: **unadjusted OR** = 4.78 (CI = 2.22-10.27), p < .001; adjusted OR* = 3.93 (CI = 1.74-8.89), p <.01; additionally adjusted OR** = 3.18 (CI = 1.32-7.65), p < .05). *adjusted for age and gender **additionally adjusted for comorbidity, cognitive impairment, depression risk and malnutrition risk.
					FES-I; **Outcome:** High CaF (≥27/64) vs. low/moderate CaF (<27/64).	Frailty vs. pre-frail or robust was associated with increased odds of high CaF: **unadjusted OR** = 5.56 (CI = 2.87-10.77), p < .001; adjusted OR* = 5.37 (CI =2.65-10.86), p < .001; additionally adjusted OR** = 3.93 (CI = 1.85-8.36), p < .001). *adjusted for age and gender **additionally adjusted for comorbidity, cognitive impairment, depression risk and malnutrition risk
Kendhapedi & Devasenapathy 2019	India; Community-dwelling	408 M: 176 (43.1 %) F: 232 (56.9 %)	Mean = 67.5 ± 6.6	FFI Robust vs. prefrail vs. frail **Cut-offs used:** - Robust: none of the criteria - Pre-frail: one or two criteria - Frail: three or more criteria	Short FES-I **Outcome:** Article does not report how the presence of CaF was defined.^††^ *^††^Attempts were made to clarify this point with authors.*	Greater frailty (pre-frail or frail, as per the FFI) was associated with greater odds of having CaF: Pre-frail vs. robust: **unadjusted OR** = 3.33 (CI = 2.08 – 5.33), p < 0.001; adjusted OR* = 2.79 (CI = 1.64-4.73), p < .001. Frail vs. robust: **unadjusted OR** = 19.24 (CI 9.71 – 38.12), p < 0.001; adjusted OR* = 13.26 (CI = 6.30-27.93, p < .001. *adjusted for age, gender, alcohol, living arrangement, nature of routine work and village in which the participant lived.
				FI Robust vs. frail **Cut-offs used:** - Robust: < 0.2 - Frail: ≥ 0.2 - 1.0		Greater frailty (when using the FI) was associated with increased CaF: **unadjusted OR** = 9.97 (CI = 6.37-15.57), p < .001; adjusted OR* = 7.48 (CI = 4.49 – 12.45), p < .001. *adjusted for age, gender, alcohol, living arrangement, nature of routine work and village in which the participant lived.
				TFI Frail vs not frail **Cut-offs used:** - Frail: 5-15 - Not frail: 0-4		Using the TFI, frailty was associated with increased CaF: **unadjusted OR** = 7.14 (CI = 4.81 – 10.58), p < .001; adjusted OR* = 5.14 (CI = 3.28 – 8.08), p < .001. *adjusted for age, gender, alcohol, living arrangement, nature of routine work and village in which the participant lived.
Merchant et al., 2020	Singapore; Community-dwelling	493 M: 102 (20.7 %) F: 391 (79.3 %)	Mean = 73.0± 8.0	5-item FRAIL scale Robust vs. prefrail + frail **Cut-offs used:** - Robust < 1 - Pre-frail: scores 1-2 - Frail: 3-5	**CaF:** Single closed-ended question: ‘Are you afraid of falling?’ Yes; yes, a lot; no **Outcome:** Presence of CaF (answered ‘yes’ or ‘yes, a lot’) vs. absence (answered ‘no’) of CaF.	Being prefrail or frail vs robust was associated with an increased odds of CaF: adjusted OR* = 2.17 (CI = 1.26-3.73), p < .01) *adjusted for age, gender and previous falls.
					**CaF-related activity restriction:** Single yes/no closed-ended question: ‘Have you ever limited your activity due to the fear?’ **Outcome:** Presence (answered ‘yes’) vs. absence (answered ‘no’) of CaF-related activity restriction.	Being prefrail or frail vs robust was not significantly associated with increased odds of activity restriction due to CaF: adjusted OR* = 1.31 (CI = 0.62-2.78), p > .05. *adjusted for age, gender and previous falls.
Ozturk et al., 2021	Turkey; Community-dwelling	1021 M: 328 (32.1 %) F: 693 (67.9 %)	Mean = 74.9± 6.9	5-item FRAIL scale robust vs. prefrail + frail **Cut-offs used:** - Robust < 1 - Pre-frail: scores 1-2 - Frail: 3-5	Single-item yes/no question: ‘Are you afraid of falling?’ **Outcome:** Presence (answered ‘yes’) vs. absence (answered ‘no’) of CaF.	Being frail or pre-frail (compared to robust) was not significantly associated with greater odds of CaF: adjusted OR* = 0.8 (CI = 0.4-1.5), p = .40). *adjusted for age, gender, falls within the last year, probable sarcopenia, sarcopenia screening, timed up and go score, usual gait speed, undernutrition, anxiety screening, urinary incontinence, sleep problems, at least one limitation in ADL, chronic pain, educational level, number of drugs and chronic diseases.
Perez-Ros et al., 2020	Spain; Community-dwelling	564 M: 208 (36.9 %) F: 356 (63.1 %)	Mean = 76.1 ±4.0	FFI Robust vs. prefrail + frail **Cut-offs used:** - Robust: none of the criteria - Pre-frail: one or two criteria - Frail: three or more criteria	Modified FES-I **Outcome:** High (≥20) vs. low (<20) CaF.	Being prefrail or frail compared to robust was associated with increased odds of CaF: adjusted OR* = 2.83 (CI = 1.54-5.18), p<0.001^††^ *adjusted for age group (80 years or more), sex and presence of previous falls. *^††^OR results with CaF as the predictor were obtained from the author on request*
Valsecchi et al., 2023	Spain and Netherlands; Community-dwelling	1080 M: 503 (46.6 %) F:543 (50.6 %); data not provided for 2.8 %	Mean = 79.4 ± 6.9	TFI Analysed as a continuous variable	FES-I **Outcome:** Analysed as a continuous variable (higher scores indicate greater CaF)	In a linear regression, greater TFI scores (increased frailty) predicted higher CaF (greater FES-I scores); with each point increase on the TFI (range from 0-15) associated with a 0.59-point increase on the FES-I in the adjusted analysis. **unadjusted β coefficient** = 0.63 (CI = 0.58 – 0.68), p < .0001 adjusted β coefficient* = 0.59 (CI = 0.53 – 0.64), p < .0001 *adjusted for age and gender

OR = Odds Ratio. ADL = activities of daily living. CaF = Concerns about falling. CFS = Clinical Frailty Scale. FES-I = Falls Efficacy Scale-International. FFI = Fried Frailty Index. FI = Frailty Index. SPPB = Short Physical Performance Battery. TFI = Tilburg Frailty Indicator.

**Table 3:** Outcomes from prospective studies.

Study	Location & Setting	Sample size; Sex (M) and (F)	Age (yrs)	Frailty assessment tool and cut-offs used in analysis	Concern about Falling (CaF) assessment tool and cut-offs used in analysis	Length of follow-up	Main findings: With frailty status/level as the predictor, CaF presence/severity as the outcome variable, odds ratios (OR) (adjusted and unadjusted), 95 % confidence interval (CI), p value
Curcio et al., 2020	Canada, Albania, Brazil, Colombia; Community-dwelling	1434 M: 681 (47.5 %) F: 753 (52.5 %)	Range= 64-75	FFI Robust vs. prefrail vs. frail **Cut-offs used:** - Robust: none of the criteria - Pre-frail: one or two criteria - Frail: three or more criteria	FES-I **Outcome:** Newly formed CaF (Score > 20) vs No CaF (Score 16-19)	24-months	Greater frailty (either frail or pre-frail as determined by the FFI) at T1 associated with greater odds of newly formed CaF at follow-up: Pre-frail vs. robust: **unadjusted OR** = 2.25 (CI = 1.72-2.95), *p* < .001 Frail vs. robust: **unadjusted OR** = 12.4 (CI = 7.6**-**20.1), *p* < .001 *Note, whilst adjusted ORs are provided, these are provided in the context of a* c*onditional inference classification tree, in which different outcomes ‘split’ the tree at different levels (making it difficult to know what precise covariates the reported ORs are controlled for). Consequently, only the unadjusted outcomes are extracted due to these having greater comparability to the analyses presented in the other studies.*
Ellmers et al., 2023	England; Community-dwelling	543 M: 262 (48.3 %) F: 281 (51.7 %)	Mean= 81.7 ± 4.63 (range = 75-98)	FFI Analysed as a continuous variable	Single-item question assessing CaF-related activity restriction: ‘Do concerns about falling stop you going out-and-about?’ **Outcome:** New onset of CaF-related activity restriction at follow-up. (I.e., answering ‘no’ to the above question at T1, but answering ‘yes’ at T2).	12-months	Higher FFI scores (i.e. greater frailty) at T1 had greater odds of developing new onset of CaF-related activity restriction at follow-up: adjusted OR* = 1.58 (CI = 1.09-2.30), p = .017. *adjusted for age, gender, ADL independence, previous falls, timed up and go, grip strength, cognitive function (Montreal Cognitive Assessment), depression (Geriatric Depression Scale), resilience (Brief Resilience Scale), generalised self-efficacy (General Self-Efficacy Scale).
Makino et al., 2021	Japan; Community-dwelling	2469 M: 1194 (48.4 %) F: 1275 (51.6 %)	Mean = 71.1 ± 4.7	FFI Robust vs. prefrail + frail **Cut-offs used:** - Robust: none of the criteria - Pre-frail: one or two criteria - Frail: three or more criteria	Single-item question: ‘Are you afraid of falling?’ **Outcome:** CaF (answered ‘very much’ or ‘somewhat’ to above question) vs. No CaF (answered ‘a little’ or ‘not at all’)	48-months	Frail or pre-frail vs. robust associated with greater odds of new onset of CaF at follow-up: **unadjusted OR** = 1.43 (CI = 1.15-1.79), p = .002; adjusted OR* = 1.33 (CI = 1.05–1.69), p = .019; additionally adjusted OR** = 1.33 (CI = 1.04-1.69), p = .022 *adjusted for age, sex, hypertension, diabetes mellitus, heart disease, pulmonary disease, knee osteoarthritis, prescribed medication, pain, MMSE and GDS. **additionally adjusted for fall experience.
Mo et al., 2023	USA; Community-dwelling	5829 M: 2449 (42.0 %) F: 3380 (58.0 %)	Mean = 77.3 ± 7.7	FFI Robust vs. pre-frail; robust vs frail **Cut-offs used:** - Robust: none of the criteria - Pre-frail: one or two criteria - Frail: three or more criteria	Single-item questions: ‘Have you been worried about falling in the last month?’ and ‘Does this worry ever limit your activities?’ **Outcome:** CaF+AR (answered (answered Yes to both questions) CaF without AR (answered Yes to first question and No to second) No CaF (answered No to both questions)	12- months	Pre-frail vs. robust is associated with greater odds of CaF (both with and without AR) at follow-up: CaF+AR: minimally adjusted RRR* = 3.76 (2.83-5.00), p < .001; adjusted RRR** = 3.74 (2.80-5.00), p < .001; additionally adjusted RRR*** = 2.96 (2.17-4.05), p < .001 CaF without AR: minimally adjusted RRR* = 1.53 (1.30-1.81), p < .001; adjusted RRR** = 1.52 (1.28-1.81), p < .001; additionally adjusted RRR*** = 1.27 (1.06-1.54), p < .05 Frail vs. robust is associated with greater odds of CaF+AR at follow-up, but not CaF without AR: CaF+AR: minimally adjusted RRR* = 7.01 (5.02-9.79), p < .001; adjusted RRR** = 6.55 (4.63-9.27), p < .001; additionally adjusted RRR*** = 3.91 (2.61-5.85), p < .001 CaF without AR: minimally adjusted RRR* = 1.70 (1.33-2.16), p < .001; adjusted RRR** = 1.69 (1.30-2.19), p < .001; additionally adjusted RRR*** = 1.26 (0.94-1.69), p > .05 *adjusted for CaF at baseline. **adjusted for age, gender, race/ethnicity, education and living arrangement. ***additionally adjusted for number of activities of daily living impairments, chronic diseases, dementia, BMI, depression, pain, hospitalisation, smoking, vigorous activity, fall history.

OR = Odds Ratio. RRR = Relative Risk Ratio. ADL = activities of daily living. BMI = body mass index. CaF = Concerns about falling. CaF+AR = activity restriction due to concerns about falling. FES-I = Falls Efficacy Scale-International. FFI = Fried Frailty Index.

All adjusted (cross-sectional and prospective) analyses reported a significant association between greater frailty and CaF, except for one cross-sectional paper [32]. ORs were mostly adjusted for age, gender and previous falls, with additionally adjusted ORs including comorbidities, educational level, chronic pain, living arrangements and mental health examinations. (See Table 2, Table 3 for full list of adjusted variables for cross-sectional and prospective studies, respectively.) Adjusted ORs tended to be larger in the cross-sectional studies (ranging from 0.8 (CI = 0.4-1.5) to 13.26 (CI = 6.30-27.93), compared to prospective studies (ORs ranging from 1.33 (1.04-1.69) to 2.42 (1.69-3.47). De Roza et al. [35] were the only cross-sectional study to not include any adjusted OR values; furthermore, their unadjusted OR value were over 6 times larger than any other included study (OR = 144.78 (CI = 13.87-1512.6)). Curcio et al.’s [37] unadjusted prospective ORs for frail vs. robust (OR = 12.4 (CI = 7.6-20.1)) were also markedly larger than any other prospective ORs. Three cross-sectional [30,35,36] and two prospective studies ([37,38] conducted separate analyses comparing non-frail to increasing levels of frailty (rather than simply comparing non-frail to, for example, a combined pre-frail and frail category). All five studies reported larger ORs with increasing frailty, evidencing a dose-response relationship.

Three studies investigated the relationship between frailty and activity restriction due to CaF [31,38,40]. In a cross-sectional study, Merchant et al. [31] found no association between frailty and CaF-related activity restriction (OR = 1.31 (CI = 0.62-2.78)) when adjusted for age, gender and previous falls. In contrast, Mo et al. [38] found that both pre-frail (fully adjusted RRR = 2.96 (CI = 2.17-4.05)) and frail older adults (fully adjusted RRR = 3.91 (CI = 2.61-5.85)) were significantly more likely to experience CaF with fear-related activity restriction at follow-up (when compared to robust older adults). This was supported by Ellmers et al. [40] who found that baseline frailty was prospectively associated with the new onset of CaF-related activity restriction at follow-up (OR = 1.58 (CI = 1.09-2.30)), when adjusted for age, gender, mobility and balance, cognitive function, psychological functioning, and general physical functioning.

### 3.6 Risk of bias assessment

The table for the ROBINS-E can be found in the appendices. With respect to the cross-sectional studies, only 3 out of 8 scored low risk of bias [31,34,39]. Two studies had some risk of bias due to missing data [32,36]: either because of a lack of information [32,33] or because they reported high levels of missing data [36]. Three out of 8 cross-sectional studies scored an overall high risk of bias [30,33,35]. This was due to either not controlling for confounds in their analysis (i.e., unadjusted ORs only [35]), or for bias in the selection of the reported results (i.e., not reporting the cut-offs used for their outcome measure [30,33]). The ROBINS-E for the prospective studies showed that 1 out of the 4 were at high risk of bias: 1 study scored high risk of bias for not controlling for confounds in their analysis (i.e., unadjusted ORs only) [37]. The other 3 prospective studies were rated as having a low risk of bias.

## 4 Discussion

**Fig. 2:**
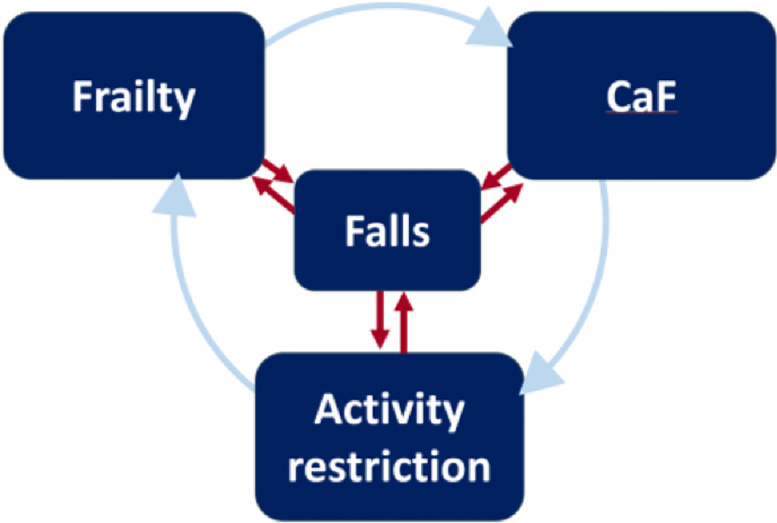
Bi-directional relationship between fraily and CaF in older adults. Frailty can cause an older person to recognise their vulnerability for experiencing a fall, which can then lead to CaF. CaF can then also lead to activity restriction, which in turn can lead to deconditioning and further physical frailty.

Our analyses revealed that greater frailty was associated with increased CaF in both cross-sectional and prospective studies; even after controlling for confounding factors. This supports the idea of a bi-directional relationship where frailty can both contribute to and result from CaF. For instance, increasing frailty at baseline was consistently associated with greater CaF and related activity restriction at follow-up, with stronger associations observed when comparing frail versus pre-frail individuals (i.e., a dose-response relationship). The results were consistent across different study designs, populations (including data from 13 different countries) and varied methods for assessing frailty and CaF, with strong associations found in most studies (e.g., ORs > 2.0 and often > 5.0). Previous research also reported that interventions which target frailty can reduce CaF [24,25] further indicating a close link between these two factors. While a previous review determined that CaF is a predictor for frailty [11], our findings suggest that this relationship is bi-directional: frailty appears both a cause and consequence of CaF. As outlined in Fig. 2, a frail older adult with high CaF is more likely to restrict their activities [40], resulting in deconditioning and further frailty [36], which in turn further increases their CaF [43]. This cycle can also increase the risk of falls, which can further contribute to frailty, CaF and activity restriction. This highlights the importance of early intervention to prevent older adults from entering this downward spiral.

The finding that increasing frailty is associated with increased CaF corroborates the views expressed by older adults themselves. Their perspective of recognition of the ‘ageing body’ and increased vulnerabilities to falls are seen as an ‘inevitable’ part of the ageing process, leading to concerns about falling and a curtailment of activities [22]. Some older adults recall experiencing a sense of dissociation between ‘mind’ and body, where their physical limitations and dependency prevent them from being able to engage in the activities that are meaningful to them. This leads to an increased sense of physical weakness and vulnerability, causing a lack of trust and confidence in themselves which results in CaF and a reduction in daily activities [44].

The use of the physical frailty phenotype (FFI) in most studies suggests that CaF could also be considered a possible subcategory of social frailty. Age, gender and a history of previous falls have been established as previous risk factors for CaF [15,45]. A number of studies included in the present review also included these risk factors in adjusted models, along with frailty. Comparisons between the ORs reported in individual studies included in the review showed that the relationship between frailty and CaF was stronger than age in 5 out of 7 studies [23,31,34,37,40]; stronger than gender in 3 studies out of 6 studies [31,36,40]; and stronger than history of previous falls in 4 out of the 6 studies [23,31,36,37]. This reinforces the strength of the association between frailty and CaF, highlighting the importance of assessing for frailty when identifying older adults most at risk for developing CaF.

A total of 5 different frailty assessment tools were used in the studies included in this systematic review, with the FFI (n = 8) being the most common. Interestingly, ORs when using the CFS tended to be higher than the other frailty tools (particularly when comparing moderately frail (score 6) compared to ‘well’ (scores 1-3)) [4]. It should be noted that the only study included in this review that failed to report a significant association between frailty and CaF used the 5-item FRAIL scale [32]. However, this study used a single-item outcome to determine CaF – rather than the gold-standard recommended approach of using the FES-I [15]. Further, the included papers varied substantially with respect to the proportion of participants who were frail, pre-frail and robust, with frailty (as determined via the FFI) ranging from 8.0 % [36] to 53.6 [34]. This heterogeneity likely accounts for the broad range of ORs observed across the different studies (although the overall positive association between frailty and CaF itself was unaffected by such heterogeneity).

Although it is difficult to directly compare findings across studies due to different frailty tools and categorisations, as well as different CaF outcomes and cut-off values used, Kendhapedi and Devasenapathy [30] directly compared multiple frailty measurement tools (cross-sectionally) to explore their association with CaF. Their study included the FFI, FI and the TFI. While the presence of frailty (vs. robust) in each outcome was significantly associated with CaF, adjusted ORs were the greatest for FFI (13.26 (CI = 6.30-27.93)), compared to either the FI (OR = 7.48 (CI = 4.49-12.45)) or the TFI (OR = 5.14 (CI = 3.28-8.08)). Likewise, directly comparing different CaF outcomes across the studies is challenging for the same reasons; although Esbri-Victor et al. [34] used both the FES-I and single-item questioning and found a stronger relationship between frailty and CaF when using the FES-I (fully adjusted OR = 3.93 (CI = 1.85-8.36) vs. 3.18 (CI = 1.32-7.65) for the single-item outcome).

CaF was predominantly measured using either the full- or short- FES-I (n = 7), or unvalidated single-item questions (n = 6). The frequent use of unvalidated assessment tools used presents a challenge as they have been found to be less sensitive for determining CaF [46], particularly in frailer older adults [47]. Moreover, the World Falls Guidelines has recommended the use of either the full or shorter version of the FES-I as the gold standard for assessing CaF [15]. Despite this, the associations between frailty and CaF obtained from single-item questions were in the same direction as those between frailty and the FES-I in all papers. Nonetheless, it is recommended that, where feasible, future research and clinicians implement the validated FES-I to assess CaF (or the short 7-item version if time is limited).

Despite a clear association between increasing frailty and CaF (both cross-sectionally and prospectively), the evidence of an association between frailty and CaF-related activity restriction was more mixed in the three studies that assessed this. Whilst Merchant et al. [31] reported a lack of significant association cross-sectionally (adjusted OR = 1.31 (CI = 0.62-2.78)), both Mo et al. [38] and Ellmers et al. [40] found a significant independent association between frailty and future CaF in prospective studies. Indeed, Ellmers et al. [40] reported a significant association between frailty and the new onset of CaF-related activity restriction (adjusted OR = 1.58 (CI = 1.09-2.30)). Future work should look to further scrutinise the relationship between frailty and future CaF-related activity restriction to clarify its nature and direction.

Although this is the first systematic review that (to our knowledge) explores the association between increasing frailty and CaF in older adults, the findings should be interpreted with respect to the limitations of this review. The heterogeneity of the included studies (e.g., the use of different tools to assess both frailty and CaF, including unvalidated single-item assessments) further limits our ability to make direct comparisons between the different data presented (and prevented us conducting a meta-analysis). Further, 6 out of 12 of the included studies were rated as having either some (2 out of 12 studies) or high (4 out of 12 studies) risk of bias. Finally, the majority (8 out of 12) of the included studies were cross-sectional. As previous work has also described how CaF can also lead to frailty [11], we thus suggest a bi-directional relationship: frailty is both a cause and consequence of CaF.

Based on the findings from this review clinicians working in frailty services should consider screening for CaF, using the short 7-item FES-I (in-line with the recommendations presented within in the World Falls Guidelines [15]). Similarly, clinicians working in falls-prevention services should regularly screen for frailty to identify those most at risk for developing CaF. Targeted interventions can then be implemented. For instance, a recent systematic review of randomised controlled trials highlighted the efficacy of exercise interventions for reducing CaF in frail and pre-frail older adults [48]. Our findings also suggest that interventions targeting frailty more broadly could be effective in addressing CaF – a hypothesis supported by previous trials [24,25]. Future work should further assess the efficacy of frailty interventions in reducing CaF by including, for example, the Short FES-I as a secondary outcome measure.

## 5 Conclusion

This review identifies strong associations between increasing frailty and both CaF and associated activity restriction. This expands previous work describing the opposite association (that CaF can lead to frailty), suggesting a bi-directional relationship. As CaF can have far-reaching negative consequences (including further frailty [11]), the present findings reinforce the importance of regularly screening for CaF in older adults at risk of falling – as recommended within the World Falls Guidelines [15]. Further research should be conducted to better understand how to best intervene to address CaF in frail and pre-frail older adults.

## Funding

Funding sources: Wellcome Trust Sir Henry Wellcome Fellowship (222747/Z/21/Z) and London Interdisciplinary Social Sciences Doctoral Training Programme by the Economic and Social Research Council (ES/P000703/1), both awarded to T.J.E.

## Sponsor's role

The sponsors had no role in the design and conduct of the study; in the collection, analysis, and interpretation of data; in the preparation of the manuscript; or in the review or approval of the manuscript.

## Authors contributions

Conceptualisation: T.J.. Ellmers. Research design: T.J. Ellmers; B. Nicklen; K. Delbaere. Search strategy: B. Nicklen; T.J. Ellmers; K. Delbaere. Searches: B. Nicklen. Article Screening: B. Nicklen; T.J. Ellmers. Data extraction: B. Nicklen. Writing first draft: B. Nicklen. Writing, editing: T.J. Ellmers; K.Delbaere. Supervision: T.J. Ellmers.

## Declaration of competing interest

The authors have no conflicts of interest.

## Appendix Supplementary materials

Image, application 1

## Footnotes

1 Note, three papers ([30], [31], [32]) included participants aged 60 and above; however, as all participants were aged 60 and above and therefore met the definition of an older adult as presented within the World Falls Guidelines [15], and as the mean age of the groups were all above 65 years (67.5 ± 6.62; 73 ± 8; 74.9 ± 6.9, respectively), these papers were included in the review.
