# Supplementary material for: Is frailty associated with increased concerns about falling and activity restriction in community-dwelling older adults? A systematic review
Source: J Frailty Aging. 2025 Jan 1;14(1):100002. doi: 10.1016/j.tjfa.2024.100002 (PMC12183984; doi:10.1016/j.tjfa.2024.100002)
Supplement: Supplementary file 1 [file mmc1.docx]

**Appendices**

Appendix 1.

Search strategies per database. Searches were last performed on the 30^th^ of December 2022.

| **Medline, Ovid** | |
| --- | --- |
| Search Number | Query |
| #1 | Old* OR Exp Aged/ OR Pensioner* OR Elder* OR Exp Geriatrics OR Geriatric* |
| #2 | Fear* OR Concern* OR Anxiety OR Balance confidence OR Activity avoidance OR Activity restriction OR **Worries** |
| #3 | Fall* OR Exp Accidental fall/ OR Fear adj2 fall* |
| #4 | Exp frailty/ OR frail* |
| #5 | #1 AND #2 AND #3 AND #4 |

| **CINAHL, EBSCO** | |
| --- | --- |
| Search Number | Query |
| #1 | Old * OR MM Aged/ OR Geriatric* OR Pensioner* OR Elder* |
| #2 | Fear* OR Concern* OR Anxiety OR Balance confidence OR Activity avoidance OR Activity restriction OR Worries or MH Fear |
| #3 | Fall* OR Exp Accidental falls/ OR Fear N1 fall* |
| #4 | Exp frailty/ |
| #5 | #1 AND #2 AND #3 AND #4 |

| **EMBASE, Ovid** | |
| --- | --- |
| Search Number | Query |
| #1 | Old* OR Exp Aged/ OR Aged OR Exp Older adulthood/ OR Exp Geriatrics/ OR Geriatric* OR Elder* |
| #2 | Fear* OR Exp Fear/ OR Concern* OR Anxiety OR Balance confidence OR Activity avoidance OR Activity restriction OR Worries |
| #3 | Fall* OR Exp falling/ OR Exp **“fear of falling**” OR Fear adj2 fall* |
| #4 | Exp frailty/ OR Exp frail elderly |
| #5 | #1 AND #2 AND #3 AND #4 |

| **PsychINFO, Ovid** | |
| --- | --- |
| Search Number | Query |
| #1 | Old* OR Exp Aging/ OR Exp older adulthood OR Elder* OR Exp geriatric/ OR Geriatric* OR Pensioner* |
| #2 | Fear* OR Exp Fear/ OR Concern* OR Exp Anxiety OR Balance confidence OR Activity avoidance OR Activity restriction OR Worries |
| #3 | Fall* OR Exp falls/ OR Fear adj2 fall* |
| #4 | Frail* |
| #5 | #1 AND #2 AND #3 AND #4 |

| **Scopus** | |
| --- | --- |
| Search Number | Query |
| #1 | Old* OR “Old* adult*” OR Elder* OR Geriatric* OR Pensioner* |
| #2 | Fear* OR Concern* OR Balance confidence OR Activity avoidance OR Activity restriction OR Worries |
| #3 | Fall* OR Exp falls/ OR Fear adj2 fall* |
| #4 | Frail* |
| #5 | #1 AND #2 AND #3 AND #4 |

Appendix 2.
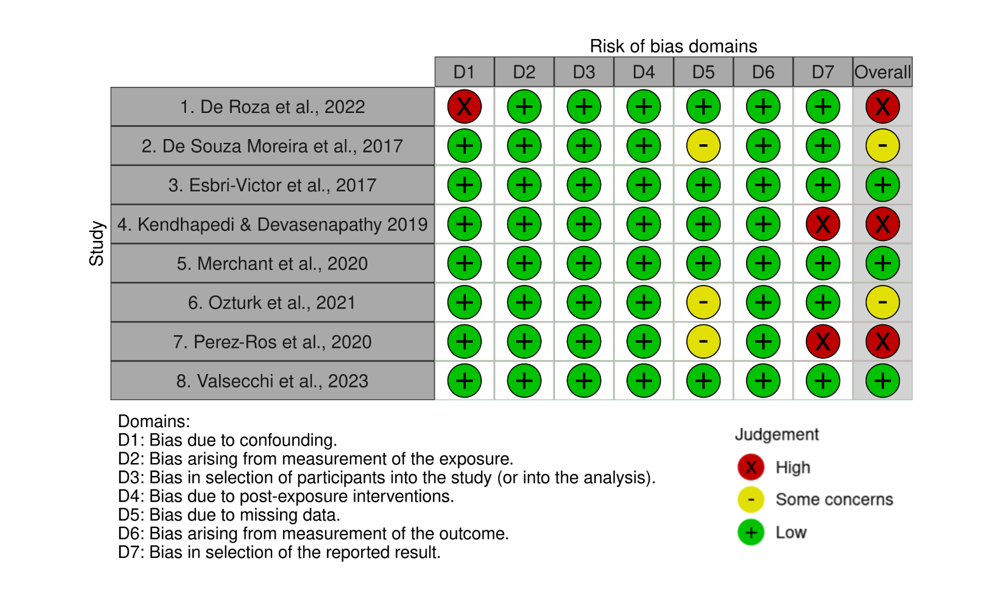
ROBINS-E table for all cross-sectional studies
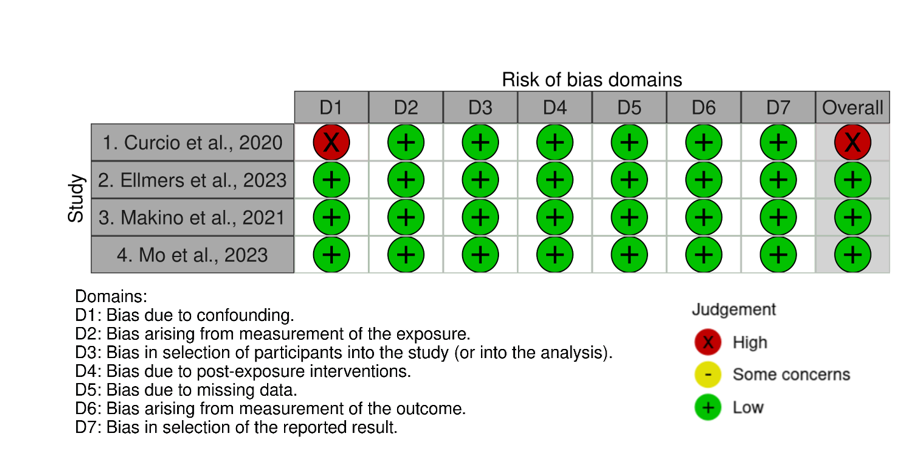


ROBINS-E table for all prospective studies
